# Is Arterial Stiffness Interconnected with Cardiovascular Drug Prescription Patterns, Body Composition Parameters, and the Quality of Blood Pressure Regulation in Hypertensive Patients?

**DOI:** 10.3390/biomedicines12092062

**Published:** 2024-09-10

**Authors:** Josipa Radić, Marijana Vučković, Hana Đogaš, Andrea Gelemanović, Andrej Belančić, Mislav Radić

**Affiliations:** 1 Department of Internal Medicine, Division of Nephrology and Dialysis, University Hospital of Split, 21000 Split, Croatia; josiparadic1973@gmail.com (J.R.); mavuckovic@kbsplit.hr (M.V.); 2 School of Medicine, University of Split, 21000 Split, Croatia; hana.dogas@gmail.com; 3 Mediterranean Institute for Life Sciences (MedILS), 21000 Split, Croatia; andrea.gelemanovic@gmail.com; 4 Department of Basic and Clinical Pharmacology with Toxicology, Faculty of Medicine, University of Rijeka, Braće Branchetta 20, 51000 Rijeka, Croatia; andrej.belancic@uniri.hr; 5 Department of Internal Medicine, Division of Rheumatology, Allergology and Clinical Immunology, University Hospital of Split, 21000 Split, Croatia

**Keywords:** arterial hypertension, arterial stiffness, pulse wave velocity, body composition, antihypertensive medication

## Abstract

Background: Arterial hypertension (AH) is a significant risk factor for cardiovascular disease and is associated with increased arterial stiffness, particularly as measured by pulse wave velocity (PWV). This study aims to explore the relationships between age groups, antihypertensive and new oral antidiabetic drugs, body composition, and arterial stiffness parameters in hypertensive patients. Methods: A single-center cross-sectional study was conducted including 584 participants who underwent 24 h ambulatory blood pressure monitoring (including central blood pressure (BP) and PWV measurement), body composition analysis, and provided medical history and current pharmacotherapy data. Results: The study found that PWV was significantly higher in patients with poorly regulated BP in those aged 65 years and older. Significant PWV predictors included systolic BP, heart rate, peripheral mean arterial pressure, peripheral pulse pressure, augmentation index, calcium channel blockers, moxonidine, sodium–glucose co-transporter 2 inhibitors, urapidil, and statin prescription. Also, statistically significant negative correlations were found between PWV and visceral fat level, fat-free mass, and the percentage of muscle mass. Conclusions: The findings suggest that arterial stiffness is interconnected with peripheral and central blood pressure parameters, body composition parameters, and prescribed hypertensive and new antidiabetic drugs.

## 1. Introduction

Arterial hypertension (AH) is one of the major risk factors for cardiovascular disease (CVD), affecting more than one-third of the population, and accounting for 20.5% of the global burden of CVD. Also, AH is the leading cause of death and disability worldwide [1]. Large artery stiffness is an established biomarker of vascular aging. The progression of artery stiffness is related not only to aging but also to cumulative exposure to many cardiovascular risk factors throughout life [2]. The relationship between AH and arterial stiffness is complex and is probably bidirectional, with high blood pressure stiffening arteries and stiff arteries inducing blood pressure (BP) increase [3].

Arterial stiffness, measured mostly by pulse wave velocity (PWV), is a crucial marker in assessing cardiovascular health. Elevated PWV is linked to worse outcomes in BP control and overall vascular function [4]. In the China Stroke Primary Prevention Trial, which included over 3000 hypertensive subjects, it was found that individuals with higher baseline PWV experienced smaller reductions in BP during short-term antihypertensive treatment. This inverse relationship between PWV and BP reduction was most pronounced for systolic BP, suggesting that arterial stiffness plays a significant role in determining the efficacy of antihypertensive therapy [5]. 

The results from a recently published meta-analysis support that antihypertensive drugs are a suitable treatment to reduce arterial stiffness in patients with AH. Based on the results, angiotensin-converting enzyme inhibitors (ACEi), angiotensin receptor blockers (ARB), beta-blockers (BB), calcium channel blockers (CCB), renin inhibitors, the thiazide diuretics/ACEi combination, the ARB/CCB combination, and the ACEi/ARB combination could be useful for patients with AH who have higher levels of arterial stiffness [6].

In addition to its role in BP control, PWV is also influenced by other cardiometabolic factors, including insulin resistance (IR) and visceral adipose tissue (VAT). The combination of IR and VAT further exacerbates arterial stiffness and increases the risk of hypertension. Notably, VAT acts as a mediator of IR’s effects on arterial stiffness, emphasizing the importance of targeting both IR and visceral fat (VF) in managing cardiovascular risk [7].

Therefore, some data suggest that antidiabetic drugs might influence arterial stiffness measured as PWV. Both glucagon-like peptide-1 receptor agonists (GLP1-RA) and, to a lesser extent, dipeptidyl peptidase 4 inhibitors (DPP4i) significantly decreased PWV [8]. Dapagliflozin can reduce arterial stiffness in people with type 2 diabetes mellitus (T2DM) [9] and is also associated with a significant decrease in BP [10] and a favorable lipid profile [10,11]. In contrast, the results from a recently published meta-analysis showed that there was no evidence of a favorable change in arterial stiffness indices found following the administration of sodium–glucose transport protein 2 inhibitors (SGLT-2i) or GLP1-RA [12].

Body composition, including fat mass (FM) and lean mass (LM), also plays crucial roles in arterial health. Increased FM, particularly in the trunk region, is linked to higher PWV and arterial stiffness. Interestingly, there is an “arterial paradox” where higher FM may sometimes correlate with better arterial function, highlighting the complexity of these relationships [13].

Considering the significant effects arterial stiffness has on overall morbidity and mortality in hypertensive and obese patients, the aim of this study was to evaluate and further explore the interplay between age groups, antihypertensive and new oral antidiabetic drugs, body composition, and arterial stiffness parameters to gain a better understanding of the effects and possible advantages in treating high-risk hypertensive patients.

## 2. Materials and Methods

### 2.1. Study Population and Design

A single-center, cross-sectional study was conducted at the Outpatient Clinic for Arterial Hypertension, Nephrology and Dialysis Division, Internal Medicine Department, University Hospital Centre Split, Croatia, between February 2021 and June 2023. 

**Figure 1:**
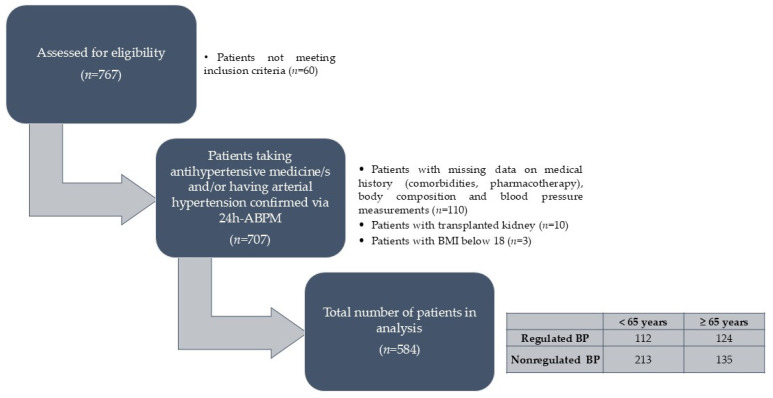
Participant selection process. Abbreviations: 24h-ABPM—24 h ambulatory blood pressure measurement; BMI—body mass index; BP—blood pressure.

Patients prescribed with antihypertensive medicine/s (AMs) and/or with confirmed AH via 24 h ambulatory blood pressure monitoring (24h-ABPM) (criteria/threshold applied: systolic blood pressure > 130 mmHg and/or diastolic blood pressure > 80 mmHg) were included if they avoided the following exclusion criteria: (i) incomplete or invalid blood pressure measurement, (ii) pregnancy, (iii) immobility, (iv) implanted pacemaker or cardioverter–defibrillator, (v) limb amputation, (vi) existing edema, (vii) existing acute infection, or (viii) active underlying malignant disease. The selection process with the inclusion and exclusion criteria is visually presented in detail in Figure 1.

All the eligible patients performed a 24h-ABPM and body composition analysis. Medical history as well as ambulatory self-reports were used to obtain data on pharmacotherapy.

The participants were informed of the purpose of the study and provided written and verbal consent. This study was conducted in accordance with the guidelines of the latest version of the Declaration of Helsinki, good clinical practice (GCP) standards, and the study protocol was approved by the Ethics Committee of the University Hospital of Split on 27 September 2022 (Class 500-03/22-01/170, Ur.no. 500-03/23-01/84).

### 2.2. Blood Pressure Measurement

BP measurement was performed based on oscillometry principles using a 24h-ABPM monitor IEM Mobile-O-Graph (IEM GmbH, Stolberg, Germany); its oscillometric cuff was placed on the non-dominant arm. The latter device was programmed to measure BP from 8:01 a.m. to 12:00 p.m. and from 12:01 a.m. to 8:00 a.m. at 15 min and 30 min intervals, respectively. The records having at least 20 ambulatory BP measurements during the day and 10 during the night (which is at least 70% of all the measurements) were considered valid. Instructions from the device manual were fully followed for all the measurements [14]. The following parameters were collected: central systolic blood pressure (cSBP; mmHg), central diastolic blood pressure (cDBP; mmHg), peripheral systolic blood pressure (pSBP; mmHg), peripheral diastolic blood pressure (pDBP; mmHg), peripheral mean arterial pressure (pMAP; mmHg), heart rate (HR; bpm), peripheral pulse pressure (pPP; mmHg), stroke volume (SV; mL/beat), augmentation index normalized to 75 beats per minute (Aix@75), PWV (m/s), and the dipping status of systolic and diastolic BP.

Data on the regulation of BP was collected using 24h-ABPM with the cut-off being systolic BP > 130 mmHg and/or diastolic BP > 80 mmHg in total 24 h average values. The regulation was defined as a good regulation of BP if both systolic and diastolic were below cut-off points, whilst the inadequate regulation of BP was differentiated as inadequate systolic, diastolic, or systolic–diastolic depending on the component/s (systolic, diastolic, or both) with elevated total 24 h average values.

### 2.3. Body Composition Measurements

On the same day when the 24h-ABPM monitor was placed, the body composition parameters were collected via the MC-780 Multifrequency Segmental Body Mass Analyzer (Tanita, Tokyo, Japan). Prior to the body composition measurements, a stadiometer was used to determine the height of the participants. All the participants were instructed in advance to follow the guidance from the instrument’s instruction manual, which are (i) emptying the bladder, (ii) not consuming food or liquid for at least 3 h before the measurement, and (iii) refraining from strenuous physical activity and alcohol consumption for at least one day before the measurement [15]. The scale, using the bioelectrical impedance model, was then used to determine the body mass (kg), body mass index (BMI; kg/m^2^), percentage of muscle mass (PMM; %), fat-free mass (FFM; kg), fat mass (FM; kg and %), visceral fat (VF), skeletal muscle index (SMI), and phase angle (PhA; °) for each study participant.

### 2.4. Medical History and Current Medication Treatment

Medical history/records as well as ambulatory self-reports were used to obtain the following data: the presence of chronic kidney disease (CKD), diabetes mellitus (DM), CVD, cerebrovascular disease (CBD), and a history of malignancy (either currently active or in remission). Additionally, the data on current pharmacotherapy were collected for the following medicine groups: BB, ACEi, ARB, CCB, diuretics, moxonidine, urapidil, α-1 antagonists, aldosterone antagonist (MRA), oral antihyperglycemics in general, SGLT-2i, GLP-1RA, insulin, statins, and uric acid inhibitors. Data on the total number of prescribed medicines as well as antihypertensive fixed-combination prescriptions were also noted.

### 2.5. Statistical Analysis

To perform descriptive statistical analysis, a Shapiro–Wilk test was first conducted to assess the normality of the numerical variables. These variables were then presented with mean and standard deviation (SD) if they were normally distributed, or with medians and interquartile range (IQR) if they deviated from normality. The categorical variables were presented with frequencies and percentages. To assess the differences between the two groups (those with regulated and nonregulated BP), the chi-square test was used for the categorical variables, and *t*-test or Mann–Whitney U test for the numerical variables depending on the normality distribution. To identify the predictors of PWV, a generalized linear regression model was performed, corrected for the effects of age, sex, the presence of DM and CKD, and BP regulation. Finally, to evaluate the correlation between PWV and visceral fat levels, the Pearson correlation analysis was performed separately. The statistically significant results were those with a *p*-value < 0.05. The regression results were presented with betas and standard errors (SEs), while the correlation results were presented with Pearson correlation coefficient (R). All the analyses were performed in R v4.3.2 [15].

## 3. Results

**Figure 2:**
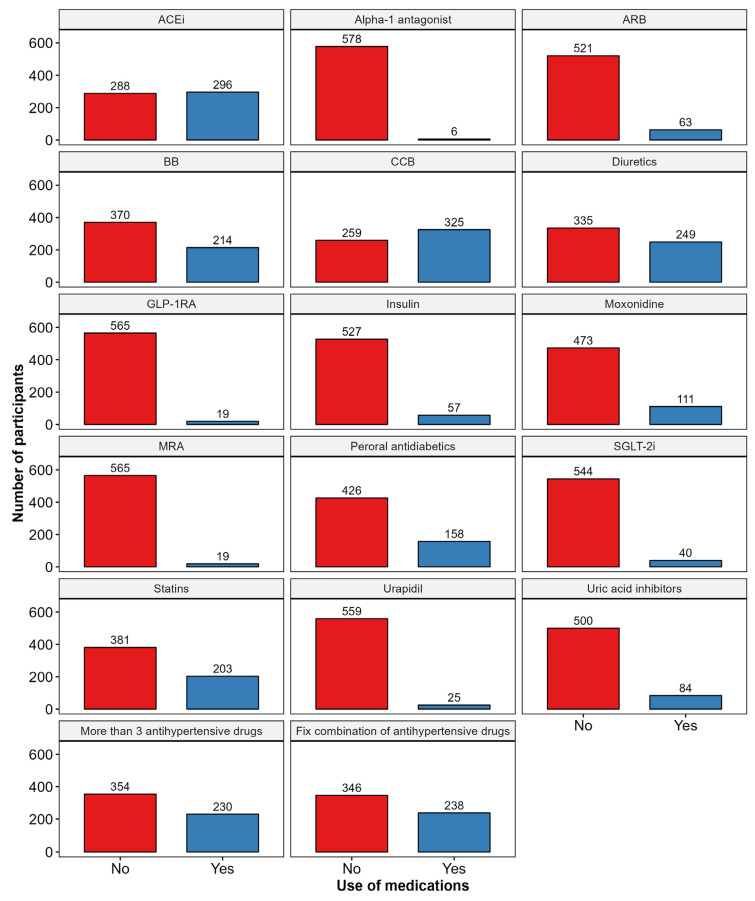
Pharmacotherapy prescription patterns in studied population. Abbreviations: ACEi—angiotensin-converting enzyme inhibitors; ARB—angiotensin receptor blocker; BB—beta-blocker; CCB—calcium channel blockers; GLP-1RA—glucagon-like peptide-1 receptor agonist; MRA—aldosterone antagonist; SGLT-2i—sodium–glucose transport protein 2 inhibitors.

A total of 584 participants (48.6% females) were included in the study, with a median age of 63 years (IQR 19). Table 1 presents the sociodemographic, body composition, and 24h-ABPM parameters. Supplementary Table S1 provides a more comprehensive overview of the general characteristics of the study population. Furthermore, Figure 2 provides a detailed overview of the pharmacotherapy prescriptions among the study participants.

To assess differences in BP regulation, the participants were classified into two groups based on their 24h-ABPM results: those with well-regulated blood pressure (systolic < 130 mmHg and diastolic < 80 mmHg) and those with poorly regulated blood pressure (systolic ≥ 130 mmHg and/or diastolic ≥ 80 mmHg). Considering age-related variations in blood pressure profiles, the participants were further divided into two subgroups: younger than 65 years and 65 years and older. Table 2 highlights significant differences in the measured parameters between these groups, while Supplementary Table S2 provides a complete overview of all the measured and evaluated parameters. Notably, both age groups exhibited higher FFM and PMM in individuals with poorly regulated blood pressure. Interestingly, PWV, the primary outcome of this study, was significantly elevated only in the older participants (≥65 years) with poorly regulated BP.

**Figure 3:**
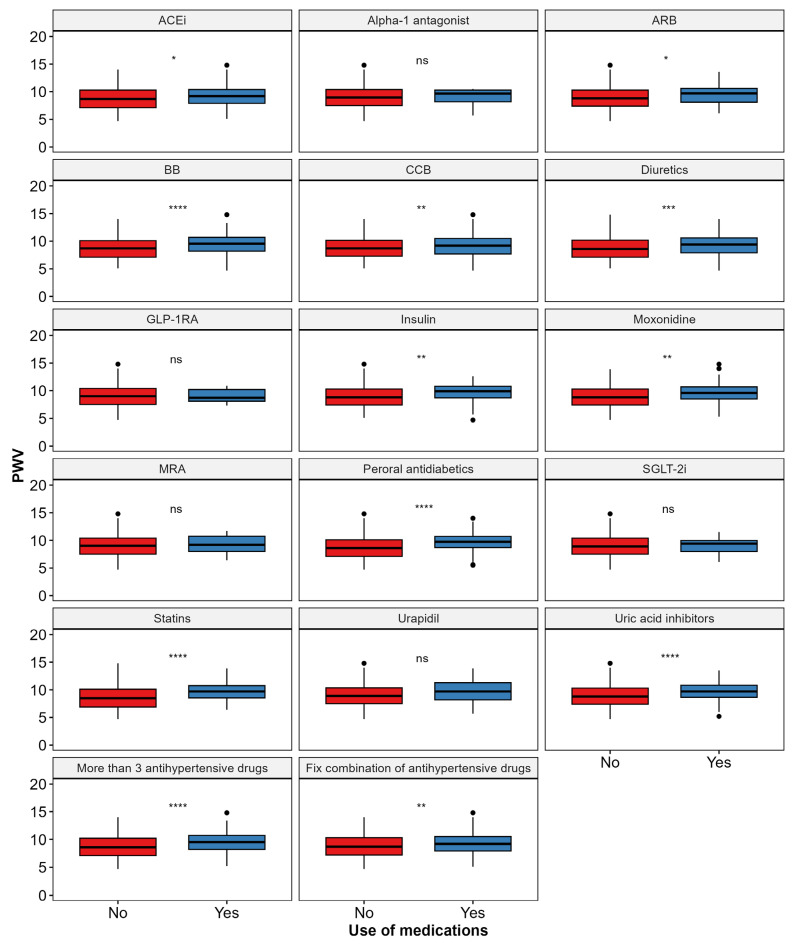
Pulse wave velocity values based on pharmacotherapy prescription patterns. Abbreviations: PWV—pulse wave velocity; ACEi—angiotensin-converting enzyme inhibitors; ARB—angiotensin receptor blocker; BB—beta-blocker; CCBs—calcium channel blockers; GLP-1RA—glucagon-like peptide-1 receptor agonist; MRA—aldosterone antagonist; SGLT-2i—sodium–glucose transport protein 2 inhibitors. *p*-value labels: **** *p* < 0.0001, *** *p* < 0.001, ** *p* < 0.01, * *p* < 0.05, ns depicts no statistical significance (*p* > 0.05).

When analyzing medication use in relation to PWV, the patients using beta-blockers BB, ACEi, and ARB had significantly higher PWV values. Other medications associated with increased PWV included CCB, moxonidine, diuretics, peroral antidiabetics, insulin, statins, and uric acid inhibitors. Additionally, PWV was significantly higher in the patients using more than three antihypertensive drugs or fixed combination drug regimens. Figure 3 presents a comprehensive analysis of the relationship between medication use and PWV.

As the primary focus of this research was to evaluate arterial stiffness, PWV was further analyzed to identify potential predictors. A generalized linear regression model was used to determine the predictors of PWV among all the study participants, adjusting for age, sex, the presence of DM and CKD, and BP regulation. After adjusting for the mentioned confounders, our regression model revealed that increased pSBP, cSBP, Aix@75, pMAP, pPP, the prescription of CCB, moxonidine, and urapidil were significantly associated with higher PWV.

Conversely, greater height, weight, VF level, FFM, PMM, HR, and the use of SGLT-2i and statins were significantly associated with lower PWV. Table 3 presents the statistically significant predictors of PWV, while Supplementary Table S3 provides a comprehensive analysis of all the predictors.

**Figure 4:**
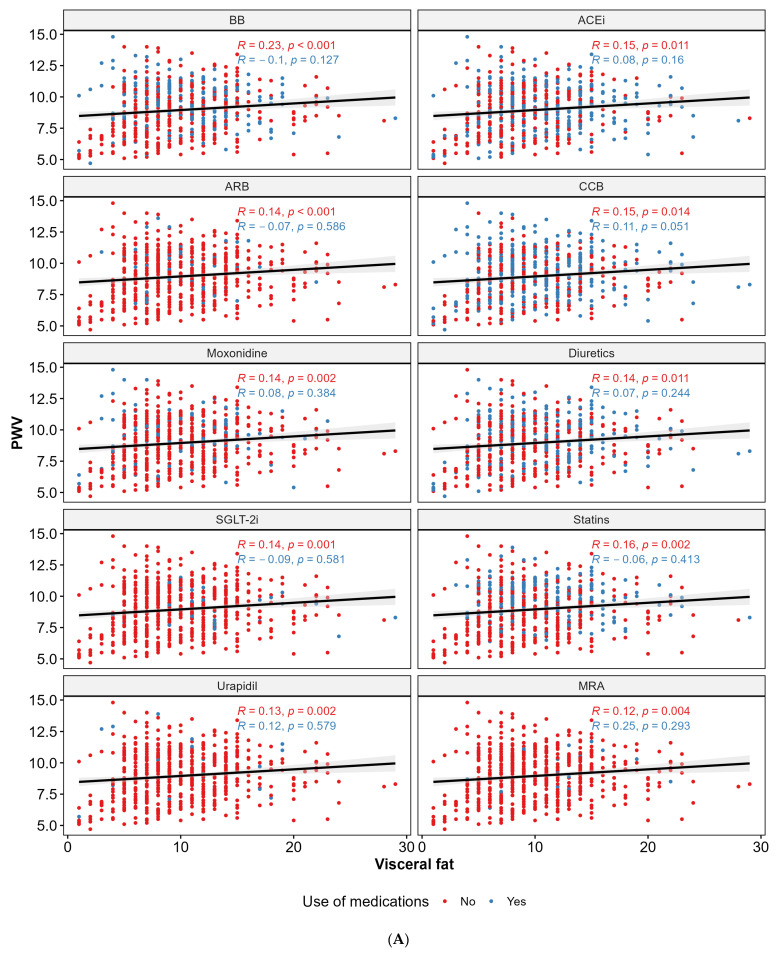

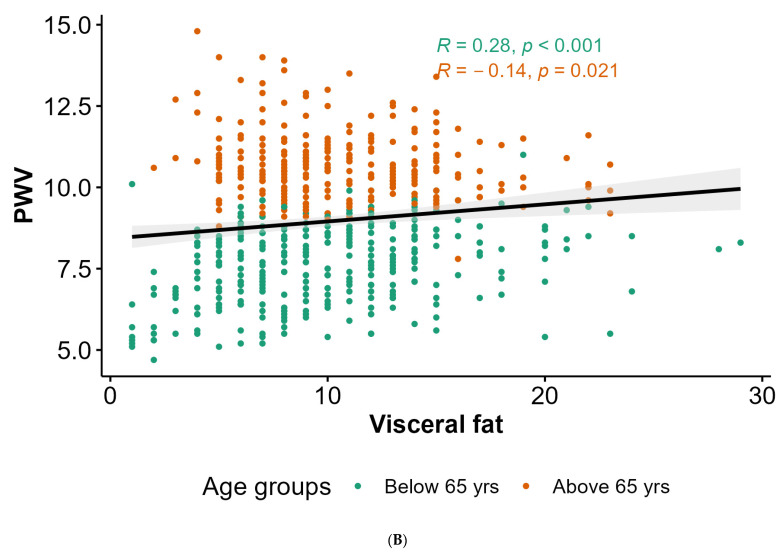
Correlations between PWV and VF level within medication groups (**A**) and age (**B**). Abbreviations: PWV—pulse wave velocity; ACEi—angiotensin-converting enzyme inhibitors; ARB—angiotensin receptor blocker; BB—beta-blocker; CCB—calcium channel blockers; MRA—aldosterone antagonist; SGLT-2i—sodium–glucose transport protein 2 inhibitors; yrs—years.

To further investigate the relationship between PWV and VF level, we analyzed the correlation between these factors while considering medication use and age. A statistically significant positive correlation between PWV and VF level was observed in the participants not using ACEi, ARB, CCB, moxonidine, SGLT-2i, MRA, diuretics, urapidil, BB, or statins. Additionally, a statistically significant positive correlation between PWV and VF level was found in the participants younger than 65 years old. Conversely, the participants aged 65 years or older exhibited a statistically significant negative correlation between PWV and VF level. Figure 4A,B illustrate these findings.

## 4. Discussion

Our research offers a thorough examination of the factors contributing to PWV in a group of hypertensive patients and highlights the complex interactions that affect arterial stiffness, including those mediated by pharmaceuticals, body composition, and hemodynamic parameters.

### 4.1. Pharmacological Therapy and PWV

Our findings suggest that the use of CCB, moxonidine, and urapidil was associated with higher PWV. This observation is particularly interesting, as CCB are generally considered to reduce arterial stiffness through their vasodilatory effects and promote vascular remodeling and improve endothelial function [6,16]. However, it is possible that the prescription of CCB. in this cohort reflects a response to higher baseline arterial stiffness or more severe hypertension or isolated systolic hypertension in this multimorbid population rather than a direct causal relationship. The association with a centrally acting antihypertensive agent moxonidine may be confounded by its use in patients with difficult-to-control AH and CKD. A positive association was also seen between PWV and urapidil, an alpha-1 adrenoceptor antagonist with central sympatholytic effects. This link may have resulted from urapidil’s use in patients who needed strong blood pressure control and had established vascular stiffness.

On the other hand, the use of SGLT-2i and statins appeared as significant negative predictors of PWV. SGLT-2i are known to offer multiple cardiorenal benefits and pleiotropic effects such as weight loss, improvement of hyperuricemia, or improvement in hepatic steatosis [17,18]. Its role in the reduction in arterial stiffness is thought to be by reducing BP, inflammation, and oxidative stress; decreasing endothelial cell activation; stimulating direct vasorelaxation; and ameliorating endothelial dysfunction or the expression of pro-atherogenic cells and molecules [18,19,20]. The negative association with PWV observed in our study adds to the growing body of evidence on SGLT-2i and vascular properties. 

Statin prescription was also negatively associated with PWV in this study. Beyond their cholesterol-lowering effects, statins have been shown to exert pleiotropic effects, including anti-inflammatory and endothelial-protective actions, which may contribute to improved arterial stiffness [21]. A recent meta-analysis found that statins had a beneficial effect on aortic arterial stiffness [22]. A study on 5105 Chinese adults with high atherosclerotic risk showed statin use associated with slower progression of arterial stiffness [23]. These findings should be interpreted while keeping in mind the design of the study and sample size.

### 4.2. Anthropometric Parameters, Body Composition, and PWV

In further analysis, the anthropometric measures of height and weight and the body composition parameters VF level, FFM, and PMM were inversely associated with PWV. Vascular stiffness and body composition have a complicated and diverse interaction. A recent study found short body height significantly associated with increasing BP and PWV measures for central and peripheral arterial stiffness in Chinese adults which is consistent with our findings [24].

The negative association with VF is particularly interesting, as visceral adiposity is typically linked to adverse cardiovascular outcomes [25]. Obesity increases arterial stiffness due to endothelial dysfunction, impaired vascular smooth muscle cell function, IR, elevated cholesterol, C-peptide levels, fat distribution, adipose tissue-related renin-aldosterone-angiotensin system, and increased leptin levels [26]. A cross-sectional population study on 2819 participants who had their VF detected by abdominal CT scan, and PWV detected by brachial ankle PWV showed similar results to ours. They found a very weak inverse association between VF and PWV but only significant in women [27]. It is possible that this finding is a specificity of our population and reflects the interplay between other epigenetic factors such as sarcopenic obesity or perhaps it could be due to the use of SGLT2i or GLP-1RA which have favorable cardiometabolic effects. 

The inverse correlation between PWV and both FFM and muscle mass, the indicators of lean body tissue, is consistent with the existing literature [28,29,30] and highlights the complex interplay between muscle mass and arterial stiffness. Arterial stiffness increases systolic BP and pulse pressure, causing muscle damage and reduced blood supply to the muscle, impairing nutrient supplementation, and leading to muscle death and reduced strength. IR, fat infiltration, inflammation, and oxidative stress contribute to arterial stiffness. Regular physical activity improves endothelial function, reduces oxidative stress, and improves muscle mass and strength, ultimately reducing aortic stiffness [30]. Also, sarcopenic obesity was found to be an even greater risk for arterial stiffness than sarcopenia or visceral obesity alone [31]. Maintaining lean body mass may thus serve as a protective factor against the development of arterial stiffness, which emphasizes the importance of promoting physical activity and resistance training in the treatment of AH.

### 4.3. Blood Pressure, Hemodynamic Parameters, and PWV

Out of BP and hemodynamic parameters, pSBP, cSBP, AIx@75, pMAP, and pPP were found to be the positive predictors of PWV while AIx@75 was found to be a negative predictor. AH and arterial stiffness are closely related, with each having a major impact on the other [32]. There is a reciprocal association between arterial stiffness and BP. The artery’s ability to dilate and constrict in response to changes in BP diminishes as arterial stiffness rises. This decreased elasticity over time may cause pSBP and pPP to rise. This is due to the fact that stiffer arteries have a reduced capacity to expand [32,33]. 

However, HR was found to be a negative predictor of PWV in our population. On the contrary, in the results of the Corinthia study, HR and PWV show a significant association independent of any confounding variables but only in those individuals with elevated aortic stiffness [34]. The median heart rate in our study was 70/min but we did not assess the heart rate variability which could be a confounding factor. 

### 4.4. Limitations

This study has several limitations that should be considered when interpreting the findings. The primary limitation is a cross-sectional study design which prevents us from drawing causal conclusions. Furthermore, physical activity was not assessed, which is an important factor in arterial stiffness progression and management. Also, given that the study was carried out at an outpatient clinic for AH in a tertiary facility, there is a possibility of recruitment bias as the patients referred typically to a tertiary facility have poorer disease history and risk profiles than those not referred there. Therefore, due to a referral bias, comorbidities and obesity may have an increased prevalence in the outpatient sample compared to general data. As a result, this analysis is specifically suited for the generally high-risk population with AH. These findings are to be noted in agreement with the earlier mentioned. To provide a deeper understanding of metabolic differences and their impact on FFM and PMM variations, including a more comprehensive set of biomarkers (e.g., laboratory indices such as lipid profile), should be a forte of future research.

## 5. Conclusions

The relationships between PWV and LM demonstrate the possible advantages of lifestyle modifications that enhance physical fitness and metabolic health. The results regarding pharmacotherapy, in particular the inverse relationship between arterial stiffness and SGLT-2i and statins, imply that these medications may have cardiovascular advantages apart from their primary indications. 

Our research further emphasizes the importance of an individualized approach to managing arterial stiffness and AH. The variety of the PWV predictors highlights the importance of considering the unique characteristics of each patient as well as their biochemical, hemodynamic, and pharmacological profiles when creating a treatment plan. More prospective studies are needed to determine the causal relationships underlying these associations and to identify potential therapeutic targets for reducing arterial stiffness and improving BP control. Long-term trials are necessary to examine the possibility that the class-specific effects of antihypertensive medicines on PWV could improve over time and be linked to better long-term outcomes.

Furthermore, routine PWV measurement and body composition measurements should be considered in the management of AH in order to improve risk stratification and therapy decisions. Given the significance of PWV in prognosis, it is crucial to conduct further studies to ascertain if antihypertensive medications specifically reduce PWV or if their effects are merely the result of lowering BP, which dilates the artery wall.

## Figures and Tables

**Table 1:** General characteristics of study population.

Variable *^,^**^,^***		All (*n* = 584)
Sex, *n* (%)	Male	300 (51.37)
	Female	284 (48.63)
Age (years), median (IQR)		63 (19)
Height (cm), mean (SD)		173.61 (9.75)
Weight (kg), median (IQR)		86 (23.43)
BMI (kg/m^2^), median (IQR)		28.75 (6.52)
FM (%), mean (SD)		29.03 (8.69)
FM (kg), median (IQR)		24.9 (13.6)
VF level, median (IQR)		10 (6)
FFM (kg), median (IQR)		61.35 (16.85)
PMM (%), median (IQR)		58.25 (16.05)
SMI, median (IQR)		8.42 (2.05)
PhA (°), median (IQR)		5.4 (1)
Smoking, *n* (%)	Yes	127 (21.75)
	No	457 (78.25)
CKD, *n* (%)	Yes	243 (41.61)
	No	341 (58.39)
DM, *n* (%)	Yes	188 (32.19)
	No	396 (67.81)
Malignancies, *n* (%)	Yes	66 (11.3)
	No	518 (88.7)
CVD, *n* (%)	Yes	120 (20.55)
	No	464 (79.45)
CBD, *n* (%)	Yes	16 (2.74)
	No	568 (97.26)
pSBP (mmHg), median (IQR)		129 (17)
pDBP (mmHg), median (IQR)		78 (13)
HR (bpm), median (IQR)		70 (12)
pMAP (mmHg), median (IQR)		101 (14)
pPP (mmHg), median (IQR)		50 (13)
cSBP (mmHg), median (IQR)		118 (15)
cDBP (mmHg), median (IQR)		79.5 (14)
AIx@75, median (IQR)		25 (10)
PWV (m/s), median (IQR)		9 (2.9)
SV (mL/beat), median (IQR)		70 (9)
Dipping systole, *n* (%)	No	423 (72.43)
	Yes	161 (27.57)
Dipping diastole, *n* (%)	No	346 (59.25)
	Yes	238 (40.75)

* Formatting of the first column: variable (unit of measurement), type of data provided. Note: in case no unit of measurement is provided, the variable does not require it as it is a number or an index. ** Measuring units in order of appearance: cm—centimeter; kg—kilogram; kg/m^2^—kilogram per meter square; %—percentage; °—degree; mmHg—millimeters of mercury; m/s—meter per second; bpm—beats per minute; mL/beat—milliliters per beat. *** Abbreviations in order of appearance: IQR—interquartile range; SD—standard deviation; BMI—body mass index, FM—fat mass; VF—visceral fat; FFM—fat-free mass; PMM—percentage of muscle mass; SMI—skeletal muscle index; PhA—phase angle; CKD—chronic kidney disease; DM—diabetes mellitus, CVD—cardiovascular disease; CBD—cerebrovascular disease; pSBP—peripheral systolic blood pressure; pDBP—peripheral diastolic blood pressure; HR—heart rate; pMAP—peripheral mean arterial pressure; pPP—peripheral pulse pressure; cSBP—central systolic blood pressure; cDBP—central diastolic blood pressure; AIx@75—augmentation index normalized to 75 beats per minute; PWV—pulse wave velocity; SV—stroke volume.

**Table 2:** Significant differences in the measured parameters regarding the age and blood pressure regulation.

Variable *^,^**^,^***		Regulated BP < 65 Years (*n* = 112)	Nonregulated BP < 65 Years (*n* = 213)	*p*	Regulated BP ≥ 65 Years (*n* = 124)	Nonregulated BP ≥ 65 Years (*n* = 135)	*p*
Age (years), median (IQR)		55.5 (12.25)	52 (13)	**0.001**	72 (7)	71 (8)	0.591
Height (cm), mean (SD)		173 (12.4)	176 (13)	**0.013**	170.21 (9.15)	171.25 (9.32)	0.367
FFM (kg), median (IQR)		62.25 (17.2)	64.1 (17)	**0.025**	58.2 (13.52)	61.3 (16.5)	**0.039**
PMM (%), median (IQR)		59.15 (16.4)	60.9 (16.2)	**0.025**	55.3 (12.9)	58.2 (15.7)	**0.039**
CVD, *n* (%)	Yes	21 (18.75)	21 (9.86)	**0.036**	37 (29.84)	41 (30.37)	1.000
	No	91 (81.25)	192 (90.14)	**0.036**	87 (70.16)	94 (69.63)	1.000
pSBP (mmHg), median (IQR)		120 (10)	134 (13)	**0.000**	121 (10)	138 (15)	**<0.001**
pDBP (mmHg), median (IQR)		73 (8)	86 (10)	**0.000**	69.52 (5.55)	80.23 (7.43)	**<0.001**
HR (bpm), median (IQR)		70.26 (8.57)	73.95 (9.38)	**0.001**	66.4 (8.92)	67.91 (9.31)	0.183
pMAP (mmHg), median (IQR)		94 (8)	107 (11)	**<0.001**	93.5 (8)	107 (9)	**<0.001**
pPP (mmHg), median (IQR)		47 (9.25)	48 (15)	**0.008**	50 (7)	58 (16)	**<0.001**
cSBP (mmHg), median (IQR)		110 (7.25)	123 (13)	**<0.001**	110 (10)	125 (11.5)	**<0.001**
cDBP (mmHg), median (IQR)		75 (8.25)	87 (9)	**<0.001**	71.5 (8)	82 (10.5)	**<0.001**
AIx@75, median (IQR)		22.05 (7.55)	23.39 (7.59)	0.132	26 (10)	28 (8)	0.001
PWV (m/s), median (IQR)		7.7 (1.63)	7.8 (1.9)	0.198	10.2 (1.1)	10.9 (1.35)	**<0.001**
ACEi, *n* (%)	Yes	73 (65.18)	85 (39.91)	**<0.001**	71 (57.26)	67 (49.63)	0.269
	No	39 (34.82)	128 (60.09)	**<0.001**	53 (42.74)	68 (50.37)	0.269
Statins, *n* (%)	Yes	35 (31.25)	41 (19.25)	0.022	66 (53.23)	61 (45.19)	0.243
	No	77 (68.75)	172 (80.75)	0.022	58 (46.77)	74 (54.81)	0.243
Fixed antihypertensive drugs combinations use, *n* (%)	Yes	60 (53.57)	61 (28.64)	**<0.001**	63 (50.81)	54 (40)	0.105
	No	52 (46.43)	152 (71.36)	**<0.001**	61 (49.19)	81 (60)	0.105

* Formatting of the first column: variable (unit of measurement), type of data provided. Note: in case no unit of measurement is provided, the variable does not require it as it is a number or an index. ** Measuring units in order of appearance: cm—centimeter; kg—kilogram; %—percentage; mmHg—millimeters of mercury; bpm—beats per minute; m/s—meter per secund. *** Abbreviations in order of appearance: IQR—interquartile range; SD—standard deviation; FFM—fat-free mass; PMM—percentage of muscle mass; CVD—cardiovascular disease; pSBP—peripheral systolic blood pressure; pDBP—peripheral diastolic blood pressure; HR—heart rate; pMAP—peripheral mean arterial pressure; pPP—peripheral pulse pressure; cSBP—central systolic blood pressure; cDBP—central diastolic blood pressure; AIx@75—augmentation index normalized to 75 beats per minute; PWV—pulse wave velocity; ACEi—angiotensin-converting enzyme inhibitors.

**Table 3:** Significant predictors of PWV in all study subjects (generalized linear regression model corrected for age, sex, presence of DM, CKD, and BP regulation).

Variable *	Beta	SE	*p*
Height	−0.01237	0.00407	0.002
Weight	−0.0031	0.00151	0.04
VF level	−0.02303	0.00668	<0.001
FFM	−0.00811	0.00316	0.011
PMM	−0.00851	0.00331	0.011
pSBP	0.03512	0.00193	<0.001
HR	−0.00856	0.0029	0.003
pMAP	0.03299	0.00313	<0.001
pPP	0.04143	0.00202	<0.001
Csbp	0.03213	0.00232	<0.001
AIx@75	0.02005	0.00488	<0.001
CCB use	0.11212	0.05252	0.033
Moxonidine use	0.1839	0.06752	0.007
SGLT-2i use	−0.23252	0.10858	0.033
Statin use	−0.12192	0.05876	0.038
Urapidil use	0.3763	0.12964	0.004

* Abbreviations: PWV—pulse wave velocity; DM—diabetes mellitus; CKD—chronic kidney disease; VF—visceral fat; FFM—fat-free mass; PMM—percentage of muscle mass; pSBP—peripheral systolic blood pressure; HR—heart rate; pMAP—peripheral mean arterial pressure; pPP—peripheral pulse pressure; cSBP—central systolic blood pressure; AIx@75—augmentation index normalized to 75 beats per minute; CCBs—calcium channel blockers; SGLT-2i—sodium–glucose transport protein 2 inhibitors.

## Data Availability

The raw data supporting the conclusions of this article will be made available by the corresponding author upon reasonable request.

## Supplementary Materials

The following supporting information can be downloaded at: https://www.mdpi.com/article/10.3390/biomedicines12092062/s1, Table S1: General characteristics of studied population.; Table S2: Differences in the measured parameters regarding the age and blood pressure regulation.; Table S3: Predictors of pulse wave velocity in all study subjects (generalized linear regression model corrected for age, sex, presence of DM and CKD, and BP regulation).
